# An Engineered Double Lipid II Binding Motifs-Containing Lantibiotic Displays Potent and Selective Antimicrobial Activity against Enterococcus faecium

**DOI:** 10.1128/AAC.02050-19

**Published:** 2020-05-21

**Authors:** Xinghong Zhao, Zhongqiong Yin, Eefjan Breukink, Gert N. Moll, Oscar P. Kuipers

**Affiliations:** a Department of Molecular Genetics, Groningen Biomolecular Sciences and Biotechnology Institute, University of Groningen, Groningen, The Netherlands; b Natural Medicine Research Center, College of Veterinary Medicine, Sichuan Agricultural University, Chengdu, China; c Membrane Biochemistry and Biophysics, Department of Chemistry, Faculty of Science, Utrecht University, Utrecht, The Netherlands; d Lanthio Pharma, Groningen, The Netherlands

**Keywords:** *Enterococcus*, *Lactococcus*, haloduracin, lantibiotic, lipid II, nisin

## Abstract

Lipid II is an essential precursor for bacterial cell wall biosynthesis and thereby an important target for various antibiotics. Several lanthionine-containing peptide antibiotics target lipid II with lanthionine-stabilized lipid II binding motifs. Here, we used the biosynthesis system of the lantibiotic nisin to synthesize a two-lipid II binding motifs-containing lantibiotic, termed TL19, which contains the N-terminal lipid II binding motif of nisin and the distinct C-terminal lipid II binding motif of one peptide of the two-component haloduracin (i.e., HalA1). Further characterization demonstrated that (i) TL19 exerts 64-fold stronger antimicrobial activity against Enterococcus faecium than nisin(1-22), which has only one lipid II binding site, and (ii) both the N- and C-terminal domains are essential for the potent antimicrobial activity of TL19, as evidenced by mutagenesis of each single and the double domains. These results show the feasibility of a new approach to synthesize potent lantibiotics with two different lipid II binding motifs to treat specific antibiotic-resistant pathogens.

## Abstract (precis)

Lipid II is an essential precursor for bacterial cell wall biosynthesis and thereby an important target for various antibiotics. Several lanthionine-containing peptide antibiotics target lipid II with lanthionine-stabilized lipid II binding motifs. Here, we used the biosynthesis system of the lantibiotic nisin to synthesize a two-lipid II binding motifs-containing lantibiotic, termed TL19, which contains the N-terminal lipid II binding motif of nisin and the distinct C-terminal lipid II binding motif of one peptide of the two-component haloduracin (i.

## INTRODUCTION

Lantibiotics are potent lanthionine-containing antimicrobial peptides that are ribosomally synthesized and posttranslationally modified (RiPPs) (1). The ribosomal synthesis and enzymatic modifications installed by lantibiotic enzymes provide a powerful discovery platform to develop novel antimicrobial peptides with high therapeutic potential (2). One of the best-studied lantibiotics is nisin. Its synthesis is controlled by the NisRK two-component regulatory system (3). In this system, nisin induces the nisin operon at the nisin A promoter via the NisRK signal-transduction system, thus controlling the expression of the genes coding for prenisin, the dehydratase NisB, the cyclase NisC, and the transporter NisT or any cloned gene of interest (4). The nisin biosynthetic system has a wide substrate tolerance; a variety of precursor peptides can be modified by this biosynthetic machinery when fused to the leader peptide of nisin (5–9). Therefore, this system is very well suited and convenient for engineering lantibiotics.

**FIG 1:**
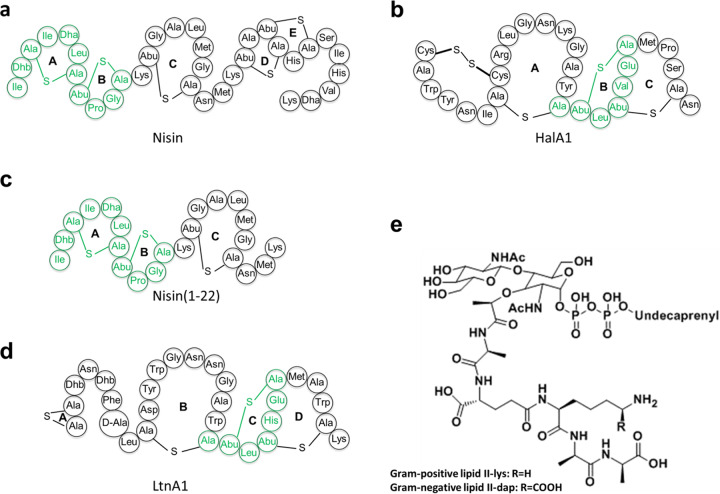
Structures of some lipid II binding lantibiotics and lipid II. Residues known to be involved in lipid II binding are colored green. (a) Nisin is a natural lantibiotic produced by various Lactococcus lactis strains. (b) HalA1 is one of the peptides of the natural two-component lantibiotic haloduracin, which is produced by Bacillus halodurans C-125. (c) Nisin(1-22) is a truncated synthetic lantibiotic derived from nisin. (d) LtnA1 is one of the peptides of the natural two-component lantibiotic lacticin 3147, which is produced by Lactococcus lactis subsp. *lactis* DPC3147. (e) Structure of Gram-positive and Gram-negative lipid II.

To engineer a lantibiotic containing two lipid II binding sites, we made use of the lantibiotics nisin, haloduracin, and lacticin A as the templates. Nisin is a 34-residue RiPP produced by various Lactococcus lactis strains (Fig. 1a). The two N-terminal rings of nisin physically interact with lipid II, resulting in formation of nisin-lipid II hybrid pores in the target membrane and inhibition of cell wall synthesis via lipid II abduction (10, 11). Haloduracin and lacticin 3147 are both two-peptide lantibiotics composed of HalA1 and HalA2 and LtnA1 and LtnA2, respectively. Both two-peptide lantibiotics have high antimicrobial potency against a range of Gram-positive bacteria (8, 12–14). Notably, the single peptides of the pair are devoid of antimicrobial activity. HalA1 and LtnA1 both contain a CTLTXEC lipid II binding motif (Fig. 1b and d). Variants with mutations in this area have reduced or even abolished antimicrobial activity (12, 13, 15, 16). The N-terminal lipid II binding site of nisin (Fig. 1a, green part) and the C-terminal lipid II binding site of HalA1 and LtnA1 (Fig. 1b and d, green parts) provide an opportunity to engineer lantibiotics with two lipid II binding motifs.

Lipid II (GlcNAc-MurNAc-pentapeptide-pyrophosphoryl-undecaprenol) (Fig. 1e) plays an essential role in the synthesis of the bacterial cell wall (11, 17). The crucial role of lipid II in cell wall synthesis makes it an excellent target for many antibiotics, including vancomycin, ramoplanin, mannopeptimycins, and teixobactin, and a number of lantibiotics, including: nisin, NAI-107, gallidermin, nukacin ISK-1, mersacidin, haloduracin, and lacticin A (11–20). A large number of potent antibiotics have the lipid II binding motif and bind lipid II in a 2:1 stoichiometry (14, 17, 19, 21–26). Only NAI-107, a very effective lantibiotic that will soon be tested in clinical trials, appears to bind in a 1:1 stoichiometry. Notably, while the short nisin(1-12) fragment is inactive, a vancomycin-nisin(1-12) hybrid is active against vancomycin-resistant enterococci (27).

As lipid II is an important target, it is of interest to study whether molecules with two different lipid II binding motifs display enhanced antimicrobial activity and/or exert altered specificity for difficult-to-treat target organisms. In this study, the nisin biosynthetic system was used to produce, modify, and secrete designed lantibiotics with two lipid II binding motifs. The following aspects of these lantibiotics were investigated: lipid II binding, bacterial killing, ability to form pores, and structure-dependent antimicrobial activity against pathogenic microorganisms. Taken together, the study demonstrates that the combination of two different lipid II binding motifs in engineered lantibiotics provides a novel and viable approach in the discovery of effective lantibiotics.

## RESULTS

### Expression of lanthipeptides with two lipid II binding motifs.

**FIG 2:**
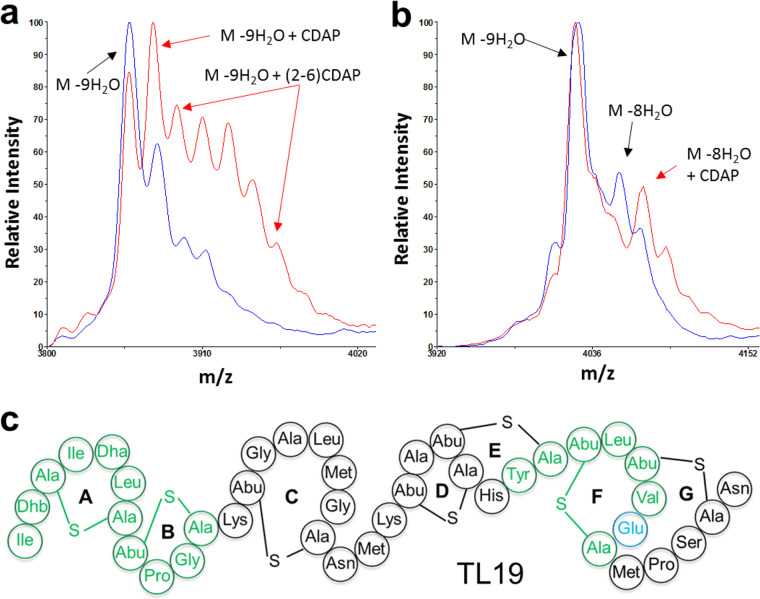
MALDI-TOF MS data of TL17 and TL19 before (blue) and after (red) treatment with CDAP. (a) TL17; (b) TL19; (c) hypothetical structure of TL19.

The designed peptides are composed of the N-terminal lipid II binding site of nisin (Fig. 1a, green part), the C-terminal lipid II binding site of either haloduracin (HalA1) or lacticin 3147 (LtnA1) (Fig. 1b and d, green parts), and appropriate linkage between these domains (Table 1). DNA sequencing confirmed the correct sequence of all 20 double lipid II binding motifs-containing peptides. All 20 designed peptides (Table 1) were produced and efficiently modified by the dehydratase NisB; more than 75% of Thr and Ser residues were dehydrated (Table 1). Two of the modified candidates (TL17 and TL19) showed potent antimicrobial activity against Micrococcus flavus (see Fig. S1 in the supplemental material), and both of these peptides were correctly dehydrated as predicted (Table 1). NisC-mediated cyclization was investigated using free cysteine-modifying 1-cyano-4-dimethylaminopyridinium tetrafluoroborate (CDAP) under reducing conditions followed by mass spectrometry (MS) analysis. No adducts were observed for the TL19 main product, indicating that all cysteines had reacted with dehydroamino acids to form (methyl)lanthionines (Fig. 2b). However, CDAP adducts were observed in TL17 (Fig. 2a), indicating that not all rings had been formed. These results suggest a likely TL19 structure (Fig. 2c), a lantibiotic with seven thioether rings and two potential lipid II binding motifs. Mass spectrometry demonstrated TL17 and TL19 were correctly collected by high-performance liquid chromatography (HPLC) purification (see Fig. S2).

**TABLE 1:** Amino acid sequence and dehydrations of designed peptides

Peptide	Amino acid sequence^*a*^	Mass (Da)		Dehydrations (observed/predicted)^*b*^
		Predicted	Measured^*b*^	
TL1	ITSISLCTPGC*TLTVECMPSCN*	5,453.16	**5,452.5**	**6/6**
TL2	ITSISLCTPGCTLTHECMAWCK	5,578.33	5,596.12	5/6
TL3	ITSISLCTPGCKTGALM*YCTLTVECMPSCN*	6,303.22	**6,302.93**	**7/7**
TL4	ITSISLCTPGCKTGALMWCTLTHECMAWCK	6,451.53	6,469.35	6/7
TL5	ITSISLCTPGCKTGALMG*CTLTVECMPSCN*	6,197.10	**6,196.88**	**7/7**
TL6	ITSISLCTPGCKTGALMGCTLTHECMAWCK	6,322.27	6,340.12	6/7
TL7	ITSISLCTPGCKTGALMG*YCTLTVECMPSCN*	6,360.27	**6,360.54**	**7/7**
TL8	ITSISLCTPGCKTGALMGWCTLTHECMAWCK	6,508.48	6,526.11	6/7
TL9	ITSISLCTPGCK*TCRLGNKGAYCTLTVECMPSCN*	6,730.67	**6,729.89**	**7/7**
TL10	ITSISLCTPGCK*SCRLGNKGAYCTLTVECMPSCN*	6,716.65	**6,716.24**	**7/7**
TL11	ITSISLCTPGCKTDYWGNNGAWCTLTHECMAWCK	6,956.80	**6,957.34**	**7/7**
TL12	ITSISLCTPGCKSDYWGNNGAWCTLTHECMAWCK	6,942.77	6,969.10	6/7
TL13	ITSISLCTPGCKTGALMGCNMKTAG*CTLTVECMPSCN*	6,884.92	**6,884.21**	**8/8**
TL14	ITSISLCTPGCKTGALMGCNMKTAGCTLTHECMAWCK	7,010.09	7,027.98	7/8
TL15	ITSISLCTPGCKTGALMGCNMKTAG*YCTLTVECMPSCN*	7,048.09	7,065.84	7/8
TL16	ITSISLCTPGCKTGALMGCNMKTAGWCTLTHECMAWCK	7,196.30	7,214.05	7/8
TL17	ITSISLCTPGCKTGALMGCNMKTATCH*CTLTVECMPSCN*	7,151.24	**7,150.87**	**9/9**
TL18	ITSISLCTPGCKTGALMGCNMKTATCHCTLTHECMAWCK	7,276.42	7,311.80	7/9
TL19	ITSISLCTPGCKTGALMGCNMKTATCH*YCTLTVECMPSCN*	7,314.41	**7,315.06**	**9/9**
TL20	ITSISLCTPGCKTGALMGCNMKTATCHWCTLTHECMAWCK	7,462.63	7,480.25	8/9
TL19 (2Asp)	I*D*SISLCTPGCKTGALMGCNMKTATCHYCTLTVECMPSCN	7,346.67	7,364.27	7/8
TL19 (34Ala)	ITSISLCTPGCKTGALMGCNMKTATCHYCTLTV*A*CMPSCN	7,256.65	**7,257.13**	**9/9**
TL19 (2Asp, 34Ala)	I*D*SISLCTPGCKTGALMGCNMKTATCHYCTLTV*A*CMPSCN	6,334.59	6,351.82	7/8

a For peptides TL1 to TL20, the amino acids from the N terminus of nisin are underlined; the amino acids from the C terminus of haloduracin A1 are italicized; and the amino acids from the C terminus of lacticin A1 are without any special format. The mutated amino acids of TL19 mutations are underlined and italicized.

b The major assay products with predicted dehydration are in boldface font. Dehydration of Ser/Thr is the first step in the lanthionine ring formation.

### TL19 exerts high activity against antibiotic-resistant Enterococcus faecium.

The antibacterial activities of TL17 and TL19 were measured by a MIC assay. Due to its ability to form pores in the target cell membrane and to inhibit cell wall synthesis, nisin (Fig. 1a) is a very potent lantibiotic (10). Nisin(1-22) (Fig. 1c) is unable to form pores, only binds to lipid II, and thereby halts cell growth without killing the cells (28). Therefore, in the MIC assays, nisin was used as a well-known antibiotic control, and nisin(1-22) was used as a one-lipid II binding motif-containing lantibiotic control with relatively low activity. Both TL17 and TL19 displayed potent activity against Gram-positive pathogens, including difficult-to-treat enterococci (Table 2). TL17 and TL19 were much more active against most target bacteria than nisin(1-22) (Table 2), which has only one lipid II binding motif and which is devoid of pore-forming capacity (28). In particular, TL17 and TL19 displayed higher antimicrobial activity against E. faecium, Enterococcus faecalis, and Bacillus cereus. TL17 and TL19 had potent antibacterial activity against antibiotic-resistant E. faecium and had 16-fold and 64-fold higher antibacterial activity, respectively, than nisin(1-22). Moreover, TL19 had a 2- to 4-fold lower MIC value against most of the E. faecium strains tested than native nisin, which not only binds lipid II but also forms pores (11, 17). TL17 and TL19 were ineffective against most Gram-negative bacteria. However, TL19 showed antimicrobial activity against a strain of Acinetobacter baumannii. In contrast to TL19, TL17 was incompletely modified and exerted lower antimicrobial activity against the tested pathogens (Table 2 and Fig. 2a; see also Fig. S3). Therefore, TL19 was selected for further studies, in particular, on its mode of action.

**TABLE 2:** Antimicrobial activity of TL17 and TL19 against pathogenic microorganisms

Organism and type^*a*^	MIC (μM [μg/ml])^*b*^			
	Nisin	Nisin(1-22)	TL17	TL19
E. faecium LMG16003 (VRE)	1.9 (6.4)	60 (128)	3.8 (14.7)	0.9 (3.6)
E. faecium LMG11423	1.9 (6.4)	60 (128)	3.8 (14.7)	0.9 (3.6)
E. faecium ATCC E1321 (AGRE)	1.9 (6.4)	60 (128)	3.8 (14.7)	0.9 (3.6)
E. faecium ATCC TX0133a04	1.9 (6.4)	60 (128)	3.8 (14.7)	0.9 (3.6)
E. faecium ATCC TX0082 (AEKVRE)	1.9 (6.4)	60 (128)	3.8 (14.7)	0.9 (3.6)
E. faecium 4Tom19	1.9 (6.4)	60 (128)	3.8 (14.7)	0.9 (3.6)
E. faecium 6Tom18	1.9 (6.4)	30 (64)	1.9 (7.3)	0.5 (2)
E. faecium ATCC HF50105 (ETVRE)	1.9 (6.4)	30 (64)	1.9 (7.3)	0.5 (2)
E. faecium ATCC U0317 (ACRE)	3.8 (12.7)	>60 (128)	7.5 (29)	1.9 (7.6)
E. faecium ATCC E1162 (ARE)	3.8 (12.7)	>60 (128)	7.5 (29)	1.9 (7.6)
E. faecalis LMG8222	1.9 (6.4)	>60 (128)	15 (58)	7.5 (30)
E. faecalis LMG16216 (VRE)	1.9 (6.4)	>60 (128)	>15 (58)	15 (60)
E. faecalis LMGV583	1.9 (6.4)	>60 (128)	>15 (58)	7.5 (30)
S. aureus LMG10147	3.8 (12.7)	15 (32)	>15 (58)	7.5 (30)
B. cereus ATCC 10987	1.9 (6.4)	>60 (128)	15 (58)	15 (60)
B. cereus ATCC 14579	1.9 (6.4)	30 (64)	15 (58)	15 (60)
A. baumannii LMG01041	3.8 (12.7)	>60 (128)	>15 (58)	7.5 (30)
Escherichia coli GSK 12	15 (50)	>60 (128)	>15 (58)	>15 (60)

a VRE, vancomycin-resistant enterococci; AGRE, ampicillin-gentamicin-resistant enterococci; AEKVRE, ampicillin-erythromycin-kanamycin-vancomycin-resistant enterococci; ETVRE, erythromycin-tetracycline-vancomycin-resistant enterococci; ACRE, ampicillin-ciprofloxacin-resistant enterococci; ARE, ampicillin-resistant enterococci.

b The MIC was determined by broth microdilution. Nisin was used as a well-known antibiotic control, and nisin(1-22) was used as a one-lipid II binding motif lantibiotic control.

### Time-dependent killing of E. faecium by TL19.

**FIG 3:**
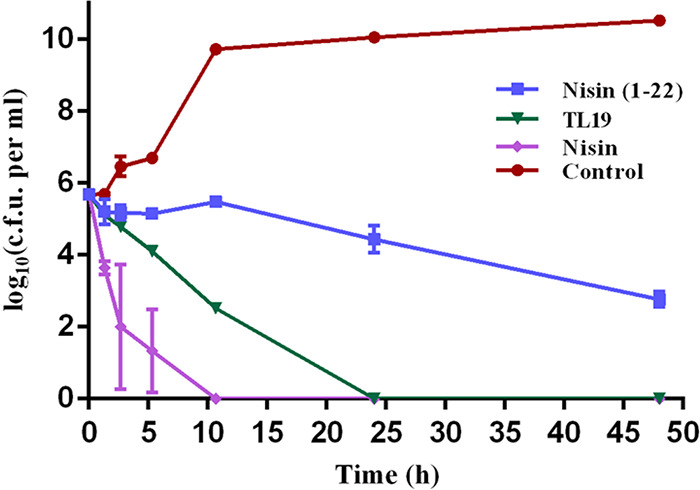
Time-dependent killing of pathogens by TL19, nisin, and nisin(1-22). E. faecium (LMG16003; vancomycin-resistant strain) was challenged with lantibiotics (10× MIC). Data are representative of 3 independent experiments ± the standard deviation (SD).

Measuring the time dependence of antibiotic action is widely used to establish whether a compound is bacteriostatic or bactericidal (22, 29). In this study, we monitored the killing kinetics of the engineered peptide TL19 and a number of other lantibiotics against vancomycin-resistant E. faecium cells. Due to its ability to form pores in the target cell membrane, together with cell wall biosynthesis inhibition, nisin is well known as a bactericidal lantibiotic (10). However, nisin(1-22) is unable to form pores and only halts cell growth without killing the cells (28). The time-dependent killing assay showed that TL19 had caused complete killing at 24 h postexposition (Fig. 3). Nisin(1-22) did not reduce the population of viable cells within 11 h postexposition, and only slight killing was observed in 48 h (Fig. 3). Nisin acted faster than TL19 and much faster than nisin(1-22), significantly reducing the population of viable cells until completely killing all cells within 11 h. The results demonstrated that TL19 not only halts cell division, like nisin(1-22), but also reduces the population of viable bacterial cells.

### TL19 does not form pores.

**FIG 4:**
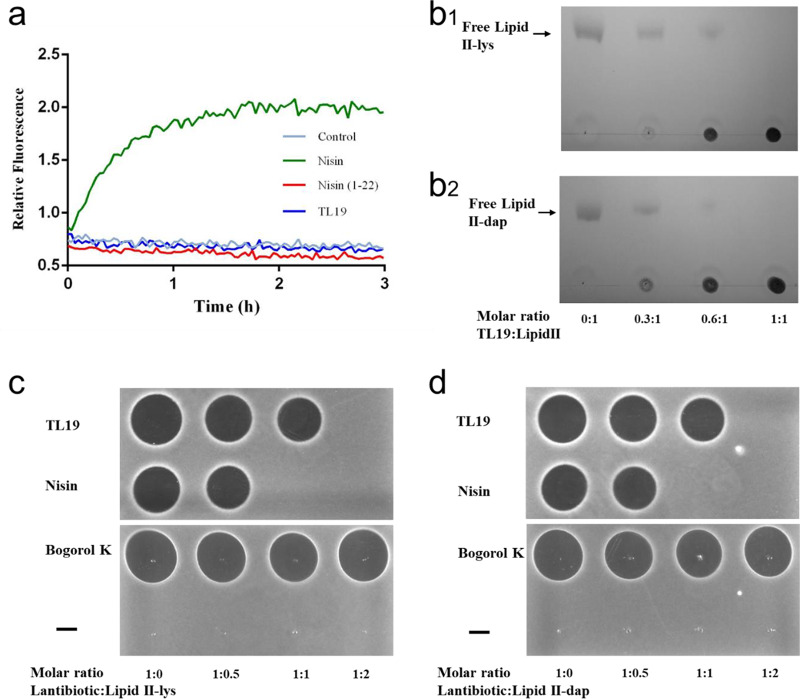
(a) E. faecium LMG16003 cells pretreated with propidium iodide were exposed to antimicrobial peptides (3 μM), and the extent of membrane leakage was visualized as an increase in fluorescence. (b) TL19 binds to cell wall precursors. Complex formation of TL19 with purified cell wall precursors at the origin are at the bottom. Binding of TL19 is indicated by a reduction of the amount of free lipid II intermediates (visible at the top on the thin-layer chromatogram). The figure is representative of two independent experiments. (c and d) Spot-on-lawn assays with E. faecium LMG16003 cells. Lipid II reduces TL19 or nisin activity by a lantibiotic/lipid II ratio-dependent reduction/disappearance of the zone of inhibition. Neither lipid II-lys nor lipid II-dap disrupted the diffusion of a non-lipid II binding peptide antibiotic, bogorol k, in the agar. Lipid II alone did not show activity.

To assess pore-forming capacity of TL19 on whole-cell membranes, a pore formation assay was performed using the dye propidium iodide (Fig. 4a). Propidium iodide is a cell membrane-impermeable nucleic acid-intercalating dye. Upon pore formation in the cell membrane, this dye enters the cell and binds to nucleic acids in the cytoplasm, causing an increase in fluorescence. Immediate pore formation was observed when E. faecium 16003 cells pretreated with propidium iodide were exposed to nisin, which not only binds lipid II but also forms pores (11, 17), whereas addition of neither TL19 nor nisin(1-22) caused any fluorescence increase, even after a relatively long period of 3 h (30). These results indicate that TL19, under the experimental conditions applied, does not form pores in the cellular membrane.

### TL19 has two functional lipid II binding domains.

The direct interaction of TL19 with Gram-positive lipid II (lipid II-lys) or Gram-negative lipid II (lipid II-dap) was investigated. Purified lipid II-lys or lipid II-dap was incubated with TL19 in various molar ratios ranging from 0.3 to 2 (TL19/lipid II). Subsequent thin-layer chromatography was used to analyze the migration behavior (Fig. 4b1 and b2). Free lipid II migrated to a defined position (Fig. 4b1 and b2, first lane). However, in complex with TL19, lipid II remained at the origin line as a clear spot (Fig. 4b1 and b2, 2nd to 4th lanes). Already at a low ratio (0.3:1) of TL19 to lipid II, lipid II migration was significantly inhibited. At a 1:1 ratio of lipid II and TL19, both lipid II-lys and lipid II-dap were fully trapped in a stable complex that prevented migration of lipid II from the origin line. Interestingly, the stoichiometry of both nisin and two-component haloduracin to lipid II reported in earlier studies is 2:1 (11, 16). Further studies were performed by a spot-on-lawn assay (Fig. 4c and d). Addition of either lipid II-lys or lipid II-dap at a 1:1 (nisin/lipid II) ratio abolished the antibacterial activity of nisin against E. faecium LMG16003. In sharp contrast to nisin, TL19 still showed potent antibacterial activity against E. faecium LMG16003 upon addition of either lipid II-lys or lipid II-dap at a 1:1 (TL19/lipid II) ratio. The antibacterial activity of TL19 against indicator strains was only abolished by further addition of lipid II resulting in a 1:2 ratio (TL19/lipid II). These results indicated that TL19 has two active lipid II binding domains.

### Both lipid II binding domains of TL19 contribute to antibacterial activity.

**FIG 5:**
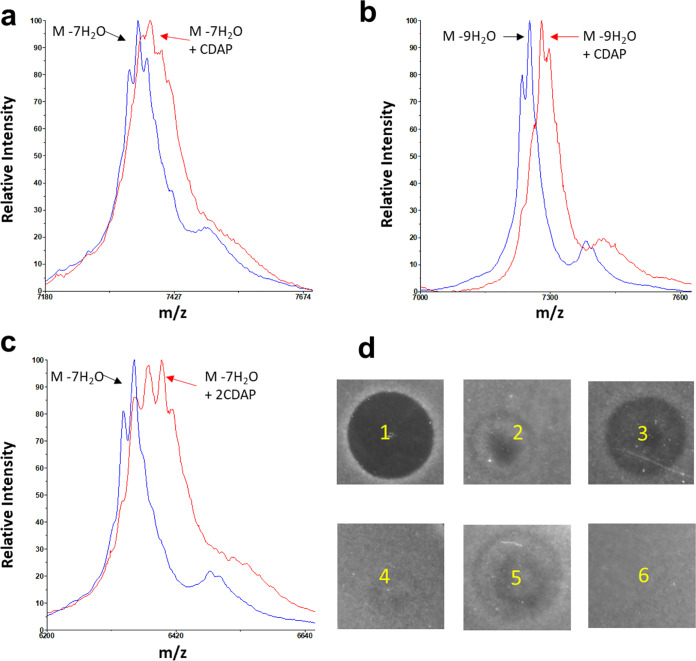
Importance of both lipid II binding motifs for antimicrobial activity against E. faecium LMG16003. MALDI-TOF MS data of TL19 or TL19 mutants before (blue) and after (red) treated with CDAP. (a) TL19 (2Asp); (b) TL19 (34Ala); (c) TL19 (2Asp, 34Ala). (d) Antimicrobial activity for TL19 mutants against E. faecium LMG16003: 1, TL19 (1 nmol); 2, TL19 (2Asp) (1 nmol); 3, TL19 (34Ala) (1 nmol); 4, TL19 (2Asp, 34Ala) (1 nmol); 5, TL19 (2Asp) (1 nmol) plus TL19 (34Ala) (1 nmol); 6, negative control (buffer).

A highly conserved glutamate in HalA1 (Glu22) is essential for binding to its target lipid II (12, 15, 16). Mutating the glutamate residue in the CTLTXEC motif to alanine or glutamine completely abolished antibacterial activity (15). Recently, we found that exchange of the Thr2 residue in nisin’s lipid II binding motif with aspartic acid or glutamic acid decreased or completely abolished nisin’s antibacterial activity (see Fig. S4). Therefore, either Glu34 in TL19 was mutated to Ala, Thr2 in TL19 was mutated to Asp, or both mutations were installed together in the same TL19 variant. Exchange of the threonine residue in TL19 (Thr2) with aspartic acid caused a loss of one dehydration in the peptide; mutation of the glutamate residue in TL19 (Glu34) to Ala did not affect the extent of dehydration (Table 1). NisC-catalyzed cyclization of the mutated peptides was investigated using CDAP treatment under reducing conditions followed by MS analysis. No adducts were observed for the TL19 main product; however, CDAP adducts were observed in all the mutated peptides (Fig. 2 and 5). Bioactivity analysis after removal of the leader peptide with the NisP leader peptidase demonstrated a strong decrease in antibacterial activity for both single-amino-acid mutant peptides and a complete abolishment of activity for the double amino acid mutant under the conditions used (Fig. 5d). Moreover, combination of the two single-mutant species in one assay did not show any synergistic antimicrobial activity. These data prove that both lipid II binding domains are important for antibacterial activity, by binding to lipid II.

## DISCUSSION

Lipid II binding lantibiotics show potent antibacterial activity by binding to lipid II and subsequently inhibiting cell wall synthesis and pore formation in the cell membrane (11, 12, 14, 20, 31). The lipid II binding motif of this class of antibiotics is essential for their potent antimicrobial activity (see Fig. S4 in the supplemental material) (13, 15). Here, we show how a lantibiotic with two different lipid II binding motifs was engineered, which showed 64-fold stronger antimicrobial activity against difficult-to-treat enterococci than the activity of a one lipid II binding motif-containing lantibiotic, nisin(1-22) (Table 2), followed by functional characterization.

Engineering of lanthipeptides has been carried out in producer strains or via heterologous expression (6, 7, 28, 32–34). In this study, the nisin biosynthesis system was used to produce 20 peptides with double lipid II binding motifs. Of the designed peptides, two showed potent antimicrobial activity against Micrococcus flavus. These two active peptides have relatively longer backbones than others, allowing the two different lipid II binding motifs to bind to different lipid II units. The MIC assay showed that TL19 has potent antibacterial activity against the human pathogen E. faecium, including the antibiotic-resistant strains. Two-component haloduracin showed better antimicrobial activity against E. faecium than against E. faecalis and Staphylococcus aureus (35), indicating that the haloduracin lipid II binding motif and its surrounding residues in TL19 might be the cause of the relatively higher target specificity to E. faecium. Moreover, the specificity toward E. faecium strains might also relate to a possibly altered makeup of the cell wall of E. faecium, but this would require additional studies, because such biochemical analysis is lacking in the literature. Importantly, the fact that TL19 has higher activity than nisin against E. faecium strains shows that the additional lipid II binding site can completely compensate for the loss of pore-forming activity (a property that nisin, haloduracin, and many other lantibiotics have). The thioether rings in lantibiotics are essential for their potent antimicrobial activity, and in general, disruption of a ring results in severe reduction or abolishment of antimicrobial activity (15, 36–38). TL19 had a 4-fold higher antibacterial activity against E. faecium than the incompletely modified TL17, which indicates that full ring formation is essential for the optimal antimicrobial activity of this two lipid II binding motifs-containing peptide. These results are consistent with previous research which showed that ring C of HalA1 is essential for the antimicrobial activity of haloduracin (15). TL19 did not show pore formation capability but showed 64-fold higher antimicrobial activity against E. faecium than nisin(1-22) (Table 2), which has only one lipid II binding site. These results highlight the key content of this study, which is the demonstration that the introduction of two lipid II binding motifs into one molecule is a novel and viable approach to engineer potent antimicrobial peptides.

Thin-layer chromatography is widely used to analyze the peptide and lipid II binding stoichiometry (14, 20, 29). Previous studies showed that both nisin and HalA1 bind the peptidoglycan precursor lipid II with 2:1 stoichiometry (11, 16). In contrast, NAI-107 binds lipid II in a 1:1 ratio (NAI-107/lipid II), which then transiently converts to a 2:1 ratio, possibly as a result of a second step of NAI-107 dimerization (20). In this study, thin-layer chromatography was used to analyze the binding stoichiometry of TL19 and lipid II. The results showed that TL19 completely immobilizes lipid II when present at 1:1 stoichiometry. Furthermore, a spot-on-lawn assay showed that the antimicrobial activity of nisin was abolished by lipid II at a 1:1 (nisin/lipid II) ratio. In contrast to nisin, to abolish the antimicrobial activity of TL19, a 1:2 (TL19/lipid II) ratio was required. These results indicate that both lipid II binding motifs in TL19 bind lipid II, which may contribute to its potent antimicrobial activity. The combination of two different lipid II binding sites in one molecule may cause an overall stronger target-binding affinity.

Mutagenesis of lantibiotics has been widely performed to improve the therapeutic potential of peptides or investigate the structure-activity relationship of new peptides (15, 32, 34, 37, 39–42). In this study, the novel lantibiotic TL19 was produced by the nisin biosynthesis system. The introduction of a T2D mutation in TL19 almost abolished the antimicrobial activity of TL19. This result is consistent with previous studies, which showed that mutant 2T to D abolished the antimicrobial activity of nisin (see Fig. S4) and that HalA1 alone did not have antibacterial activity (8). The E34A mutant of TL19 also severely reduced the antimicrobial activity of the peptide. These results are consistent with previous studies on haloduracin (8), which showed that a highly conserved glutamate in HalA1 (Glu22) was essential for binding to its target lipid II (12, 15, 16). The T2D and E34A double mutant of TL19 decreased or completely abolished the antimicrobial activity of the peptide. All of the results from these three mutants clearly demonstrate that both lipid II binding sites are essential for the potent antimicrobial activity of TL19.

In conclusion, our results show that the nisin biosynthetic system can successfully be used to engineer and produce a lantibiotic (TL19) with seven thioether rings and two lipid II binding motifs. The engineered two lipid II binding motifs-containing TL19 shows higher antimicrobial activity against specific pathogens than nisin(1-22), which has only one lipid II binding motif. In particular, TL19 shows 64-fold higher antimicrobial activity against difficult-to-treat enterococci than nisin(1-22) and 2- to 4-fold higher activity than nisin itself against E. faecium. This study provides a new approach for the biosynthesis of potent lantibiotics with two different lipid II binding motifs to treat antibiotic-resistant pathogens.

## MATERIALS AND METHODS

### General materials and methods.

Reagents used for molecular biology experiments were purchased from Thermo Fisher Scientific (Waltham, MA). Other chemicals were purchased from Sigma-Aldrich (St. Louis, MO). L. lactis NZ9000 was used as the host cell for plasmid maintenance and protein expression. Constructed plasmids were sequenced at Macrogen Inc. (Amsterdam, The Netherlands).

### Molecular biology techniques.

Oligonucleotide primers used for PCR, cloning, and sequencing in this study are provided in Table S1 in the supplemental material; all of the oligonucleotide primers were purchased from Biolegio B.V. (Nijmegen, The Netherlands). Plasmids encoding the peptides were constructed by amplifying the template plasmid using a phosphorylated downstream sense (or upstream antisense) primer and an upstream antisense (or downstream sense) primer. Phusion DNA polymerase (Thermo Fisher Scientific, Waltham, MA) was used to amplify the DNA. Self-ligation of the PCR product was carried out with T4 DNA ligase (Thermo Fisher Scientific, Waltham, MA). The electrotransformation of L. lactis was carried out as previously described using a Bio-Rad gene pulser (Bio-Rad, Richmond, CA) (43). The mutations were verified by sequencing using the PrXZ12 reverse primer.

### Expression and trichloroacetic acid precipitation of peptides.

L. lactis NZ9000 cells containing the genes of the nisin synthetic machinery *nisBTC* were electroporated with His6 peptide plasmids (50 ng), plated on GM17 agar plates supplemented with chloramphenicol (5 μg/ml) and erythromycin (5 μg/ml) (GM17CmEm), and grown at 30°C for 20 to 24 h. A single colony was used to inoculate 5 ml of GM17CmEm broth. The culture was grown overnight at 30°C and then used to inoculate 45 ml (20-fold dilution) of minimal medium. Cultures were grown at 30°C to an optical density at 600 nm (OD_600_) of 0.4. Peptide expression was induced by the addition of nisin to a final concentration of 5 ng/ml, and cultures were grown at 30°C for 3 h. Cells were harvested by centrifugation at 15,000 × *g* for 15 min, and supernatants were collected. Ice-cold 100% trichloroacetic acid (TCA) was added to the ice-cold supernatants to a final concentration of 10%, and samples were subsequently kept on ice for 1 h to precipitate peptides. Samples were then centrifuged at 10,000 × *g* at 4°C for 30 min. The precipitate was washed several times with 20 ml ice-cold acetone to remove any residual TCA. Samples were dried in the fume hood and resuspended in 0.5 ml phosphate-buffered saline (PBS).

### Tricine-SDS protein gel assay.

Peptides isolated from the supernatant were separated by Tricine-SDS gel (16%) electrophoresis and visualized by Coomassie blue staining.

### Screening the antibacterial activity of peptides by spot-on-lawn assay.

To assess the antibacterial activity of TCA-precipitated samples on a agar plate, an overnight culture of Micrococcus flavus was added to the GM17 containing 0.8% (wt/vol) agar (at 42°C), and then the mixture was poured onto the plates, 30 ml per plate. After that, 5 μl of the peptides together with 1 μl of NisP were added to the plates, and the plates were transferred to the 30°C incubator for incubation overnight.

### Expression and purification of His6-tagged peptides.

L. lactis NZ9000, containing the plasmids with the genes of the nisin synthetic machinery and of the designed peptide, was used to inoculate 50 ml of GM17CmEm broth. The culture was grown overnight at 30°C and then used to inoculate 1 liter (20-fold dilution) of GM17CmEm medium. Cultures were grown at 30°C to an OD_600_ of 0.4. Peptide expression was induced by the addition of nisin to a final concentration of 5 ng/ml, and cultures were grown at 30°C for 3 h. After centrifugation at 15,000 × *g* for 15 min, supernatants were collected, the pH of the supernatant was adjusted to 7.4, and the supernatant was filtered through a 0.45-μm membrane. Supernatants were applied to a HisTrap Excel column (GE Healthcare) equilibrated with 50 mM NaH_2_PO_4_, 300 mM NaCl, and 10 mM imidazole (pH 8.0). The flowthrough was discarded, and the column was subsequently washed with 16 column volumes (CV) of wash buffer (50 mM NaH_2_PO_4_, 300 mM NaCl, 20 mM imidazole [pH 8.0]). The peptide was eluted with 15 CV elution buffer (50 mM NaH_2_PO_4_, 300 mM NaCl, 250 mM imidazole [pH 8.0]). The eluted peptide was then applied to a Sigma-Aldrich C_18_ silica gel spherically equilibrated first with 8 CV of acetonitrile (MeCN) containing 0.1% trifluoroacetic acid followed by 8 CV of 5% aqueous MeCN containing 0.1% trifluoroacetic acid. The column was washed with 8 CV of 5% aqueous MeCN containing 0.1% trifluoroacetic acid to remove the salts. Peptide was eluted from the column using up to 8 CV of 50% aqueous MeCN containing 0.1% trifluoroacetic acid. Fractions containing the eluted peptide were freeze-dried, and the peptide was subsequently dissolved in PBS containing an appropriate amount of NisP and incubated at 37°C for 2 h to cleave off the leader peptide. The insoluble material was removed by filtration through a 0.2-μm filter, and the supernatant was purified on an Agilent 1260 Infinity HPLC system with a Phenomenex Aeris C_18_ column (250 mm by 4.6 mm, 3.6-μm particle size, 100-Å pore size). Acetonitrile was used as the mobile phase, and a gradient of 35% to 40% aqueous MeCN over 18 min at 1 ml/min was used for separation. Peptide was eluted at 37% to 38% MeCN.

### Mass spectrometry.

Matrix-assisted laser desorption ionization–time of flight (MALDI-TOF) mass spectrometer analysis was performed using a 4800 Plus MALDI TOF/TOF analyzer (Applied Biosystems) in the linear-positive mode at University of Groningen. Briefly, a 1-μl sample from HPLC or TCA precipitation was spotted on the target and dried at room temperature. Subsequently, 0.6 μl of matrix solution (5 mg/ml of α-cyano-4-hydroxycinnamic acid) was spotted on each sample. After the samples had dried, MALDI-TOF MS was performed.

### Evaluation of (methyl)lanthionine formation.

After the freeze-dried samples were dissolved in 18 μl of 0.5 M HCl (pH 3), the samples were treated with 2 μl of 100-mg/ml tris[2-carboxyethyl]phosphine in 0.5 M HCl (pH 3) for 30 min at room temperature. Subsequently, the samples were treated with 4 μl of 100-mg/ml 1-cyano-4-dimethylaminopyridinium tetrafluoroborate (CDAP) in 0.5 M HCl (pH 3). After incubation at room temperature for 2 h, the samples were desalted by C_18_ ZipTip (Millipore) and analyzed by MALDI-TOF MS (42, 44).

### MIC.

MIC was evaluated by broth microdilution according to the standard guidelines (45). Briefly, the test medium was cation-adjusted Mueller-Hinton broth (MHB). Cell concentration was adjusted to approximately 5 × 10^5^ cells per ml. After 20 h of incubation at 37°C, the MIC was defined as the lowest concentration of antibiotic with no visible growth. Each experiment was performed in triplicates.

### Time-kill assay.

This assay was performed according to a previously described procedure (29). An overnight culture of cells (Enterococcus faecium LMG 16003; vancomycin-resistant strain) was diluted 50-fold in MHB and incubated at 37°C with aeration at 220 rpm. Bacteria were grown to an OD of 0.5, and then the cell concentration was adjusted to ≈5 × 10^5^ cells per ml. Bacteria were then challenged with nisin (20 μM), nisin(1-22) (600 μM), or TL19 (10 μM) in culture tubes at 37°C and 220 rpm. (peptides at 10× MIC, a desirable concentration at the site of infection). Bacteria not treated with peptides were used as a negative control. At desired time points, 100-μl aliquots were taken, centrifuged at 8,000 × *g* for 2 min, and resuspended in 100 μl of MHB. Ten-fold serially diluted samples were plated on Mueller-Hinton agar (MHA) plates. After incubating at 37°C overnight, colonies were counted and CFU per milliliter was calculated. Each experiment was performed in triplicates.

### Propidium iodide assay for membrane pore formation.

The excitation and emission wavelengths on the fluorescence spectrometer were adjusted to 533 nm and 617 nm, respectively. E. faecium LMG16003 was grown to an OD_600_ of 0.6. To this cell suspension, propidium iodide (final concentration, 2.5 μg per ml) was added and incubated for 5 min. Peptides were added to a final concentration of 3 μM. Fluorescence was monitored for 3 h, with the peptide added after ∼60 s. Representative examples from three technical replicates are shown in the figures.

### Analysis of complex formation of TL19 with lipid II by thin-layer chromatography.

Purified lipid II-lys or lipid II-dap (4 nmol) was incubated in chloroform-methanol-water (2:3:1), in the presence of increasing TL19 concentrations (TL19/lipid II molar ratios ranging from 0.3 to 2:1) in a total volume of 30 μl. After incubating for 30 min at 25°C, the mixture was analyzed by thin-layer chromatography using chloroform-methanol-water-ammonia (88:48:10:1), and the spots were visualized by iodine vapor (20, 26).

### Spot-on-lawn assay to measure TL19-lipid II complex formation.

An overnight cultured E. faecium LMG16003 was added to 0.8% MHA (wt/vol; temperature, 42°C) at a final concentration of 0.1% (vol/vol), and then the mixture was poured onto the plates (30 ml for each). Binding of peptide and lipid II was further evaluated by incubating purified lipid II with peptide in various molar ratios ranging from 0.5 to 2 with respect to the peptides (1 nmol). Subsequently, a spot-on-lawn assay was used to analyze the antimicrobial activity. After the lipid II solution drops with peptide had dried, the plates were transferred to the 37°C incubator for overnight incubation.

### Data availability.

The authors declare that all the data supporting the findings of this study are available within the paper and its supplemental material. Additional raw data are available from the corresponding author upon reasonable request.

## Supplementary Material

Supplemental file 1
