# Supplementary material for: An Engineered Double Lipid II Binding Motifs-Containing Lantibiotic Displays Potent and Selective Antimicrobial Activity against Enterococcus faecium
Source: Antimicrob Agents Chemother. 2020 May 21;64(6):e02050-19. doi: 10.1128/AAC.02050-19 (PMC7269505; doi:10.1128/AAC.02050-19)
Supplement: Supplemental file 1 [file AAC.02050-19-s0001.pdf]

Supplemental material

An engineered double lipid II binding motifs-containing lantibiotic displays potent and selective antimicrobial activity against *Enterococcus faecium*

Running title: Lantibiotic with two lipid II binding motifs

Xinghong Zhao [1, 2], Zhongqiong Yin [2], Eefjan Breukink [3], Gert N. Moll [1, 4], Oscar P. Kuipers [1,*]

[1] Department of Molecular Genetics, Groningen Biomolecular Sciences and Biotechnology Institute, University of Groningen, Groningen, 9747 AG, The Netherlands.

[2] Natural Medicine Research Center, College of Veterinary Medicine, Sichuan Agricultural University, Chengdu, 611130, China.

[3] Membrane Biochemistry and Biophysics, Department of Chemistry, Faculty of Science, Utrecht University, Utrecht, 3512JE, Netherlands.

[4] Lanthio Pharma, Rozenburglaan 13 B, Groningen, 9727 DL, The Netherlands.

* Correspondence: o.p.kuipers@rug.nl (Oscar P. Kuipers)

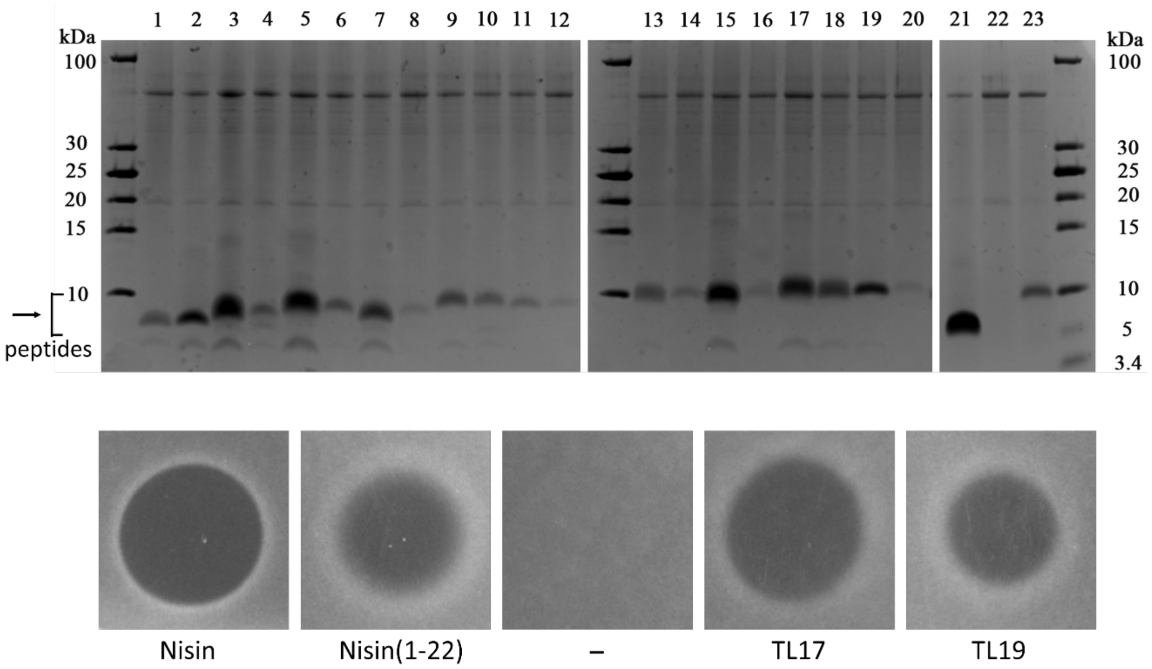

FIG S1 a, expression of peptides measured by SDS-tricine gel, 1-24 lanes: TL1-TL20, lane 21: nisin (1-22), lane 22: empty plasmid, lane 23: nisin. b, antimicrobial activity of peptides against *Micrococcus flavus.* TL1-16, TL18 and TL20 did not show antimicrobial activity against *Micrococcus flavus* (Data not shown).

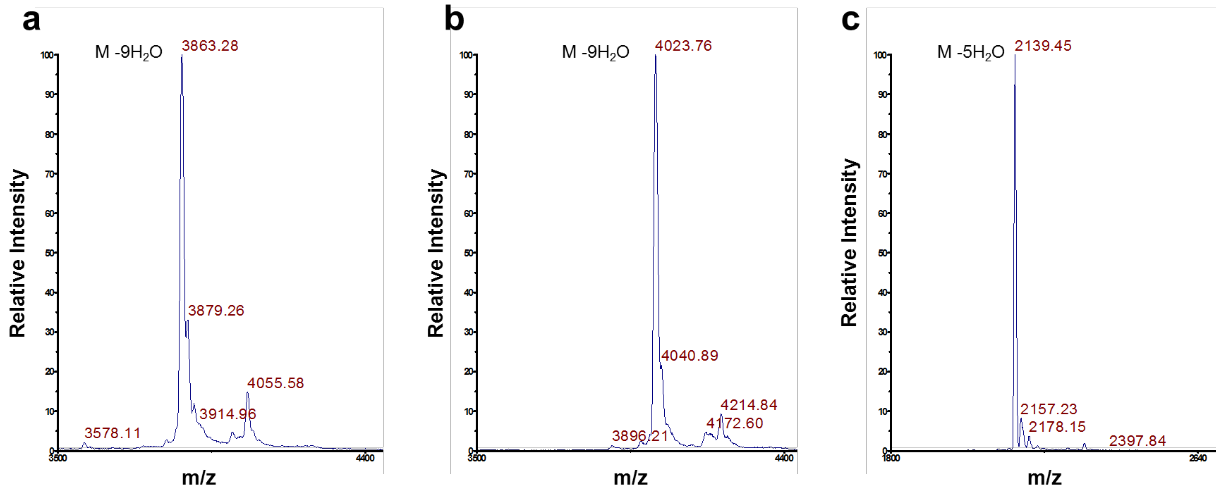

FIG S2 MALDI-TOF MS of HPLC-purified TL17 (a), TL19 (b) and nisin(1-22) (c).

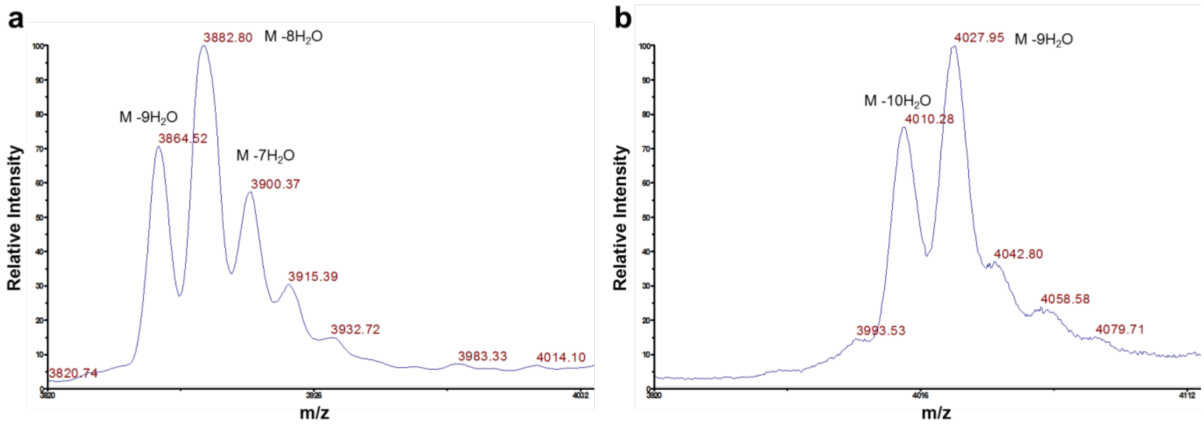

FIG S3 MALDI-TOF MS of TL17 and TL19. His-tag column-purified samples after removal of the leader were analyzed by MALDI-TOF MS.

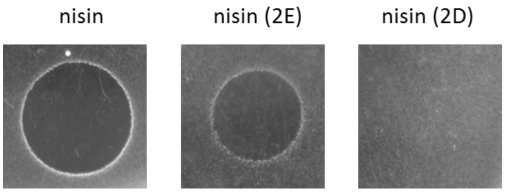

FIG S4 Antimicrobial activity of nisin, nisin (2E) and nisin (2D) against *M. flavus*.

Table S1 Primers for PCRs used in this study.

Mutants	Templates	primers	Nucleic acid sequences (5' to 3')	Characteristics (5'-lable)
Nisin(1-22)	pNZnisA-E3-his2	PZ 3	AAGCTTTCTTTGAACCAAAATTAGAAAACCAAG	5'- phosphorylation
		PZ 4	ATTTCATGTTACAACCCATCAGAGCTC	
pNZnisA-E3-his2	pNZnisA-E3	PZ 1	CATCACCATAGTACAAAAGATTTTAACTTGGATTTGGTATCTG	5'- phosphorylation
		PZ 2	GTGATGATGCATGGTGAGTGCCTCCTTATAATTTATTTTG	
TL1	pNZnisA-E3-his2	PZ 5	AATGTATGCCAAGTTGTAACTAAGCTTTCTTTGAACCAAAATTAGAAAACCAAG	5'- phosphorylation
		PZ 6	CTACTGTCAATGTACAACCGGGTGTACATAGCGAAATAC	
TL2	pNZnisA-E3-his2	PZ 7	GAATGTATGGCTTGGTGTAAATAAGCTTTCTTTGAACCAAAATTAGAAAACCAAG	5'- phosphorylation
		PZ 8	GTGTGTCAATGTACAACCGGGTGTACATAGCGAAATAC	
TL3	pNZnisA-E3-his2	PZ 5	AATGTATGCCAAGTTGTAACTAAGCTTTCTTTGAACCAAAATTAGAAAACCAAG	5'- phosphorylation
		PZ 9	CTACTGTCAATGTACAATACATCAGAGCTCCTGTTTTACAAC	
TL4	pNZnisA-E3-his2	PZ 7	GAATGTATGGCTTGGTGTAAATAAGCTTTCTTTGAACCAAAATTAGAAAACCAAG	5'- phosphorylation
		PZ 10	GTGTGTCAATGTACACCACATCAGAGCTCCTGTTTTACAAC	
TL5	pNZnisA-E3-his2	PZ 5	AATGTATGCCAAGTTGTAACTAAGCTTTCTTTGAACCAAAATTAGAAAACCAAG	5'- phosphorylation
		PZ 11	CTACTGTCAATGTACAACCCATCAGAGCTCCTGTTTTACAAC	
TL6	pNZnisA-E3-his2	PZ 7	GAATGTATGGCTTGGTGTAAATAAGCTTTCTTTGAACCAAAATTAGAAAACCAAG	5'- phosphorylation
		PZ 12	GTGTGTCAATGTACAACCCATCAGAGCTCCTGTTTTACAAC	
TL7	TL5	PZ 5	AATGTATGCCAAGTTGTAACTAAGCTTTCTTTGAACCAAAATTAGAAAACCAAG	5'- phosphorylation
		PZ 13	CTACTGTCAATGTACAATAACCCATCAGAGCTCCTGTTTTACAAC	
TL8	TL6	PZ 7	GAATGTATGGCTTGGTGTAAATAAGCTTTCTTTGAACCAAAATTAGAAAACCAAG	5'- phosphorylation
		PZ 14	GTGTGTCAATGTACACCAACCCATCAGAGCTCCTGTTTTACAAC	
TL9	TL3	PZ 15	CCAATCTACATGTTTTACAACCGGGTGTACATAG	
		PZ 16	GAAACAAAGGAGCTTATTGTACATTGACAGTAGAATGTATGCCAAGTTG	5'- phosphorylation

Table S1 Primers for PCRs used in this study (continuing).

Mutants	Templates	primers	Nucleic acid sequences (5' to 3')	Characteristics (5'-lable)
TL10	TL3	PZ 16	GAAACAAAGGAGCTTATTGTACATTGACAGTAGAATGTATGCCAAGTTG	5'- phosphorylation
		PZ 17	CCAATCTACATGATTTACAACCGGGTGTACATAGCGAAATAC	
TL11	TL4	PZ 18	CCCAATAGTCTGTTTTACAACCGGGTGTACATAG	
		PZ 19	GAAACAATGGAGCTTGGTGTACATTGACACACGAATGTATG	5'- phosphorylation
TL12	TL4	PZ 18	GAAACAATGGAGCTTGGTGTACATTGACACACGAATGTATG	
		PZ 20	CCCAATAGTCTGATTTACAACCGGGTGTACATAGCGAAATAC	5'- phosphorylation
TL13	pNZnisA-E3-his2	PZ 5	AATGTATGCCAAGTTGTAACTAAGCTTTCTTTGAACCAAAATTAGAAAACCAAG	5'- phosphorylation
		PZ 21	CTACTGTCAATGTACATCCTGCTGTTTTCATGTTACAACCCATCAGAG	
TL14	pNZnisA-E3-his2	PZ 7	GAATGTATGGCTTGGTGTAAATAAGCTTTCTTTGAACCAAAATTAGAAAACCAAG	5'- phosphorylation
		PZ 22	GTGTGTCAATGTACATCCTGCTGTTTTCATGTTACAACCCATCAGAG	
TL15	pNZnisA-E3-his2	PZ 5	AATGTATGCCAAGTTGTAACTAAGCTTTCTTTGAACCAAAATTAGAAAACCAAG	5'- phosphorylation
		PZ 23	CTACTGTCAATGTACAATATCCTGCTGTTTTCATGTTACAACCCATCAGAG	
TL16	pNZnisA-E3-his2	PZ 7	GAATGTATGGCTTGGTGTAAATAAGCTTTCTTTGAACCAAAATTAGAAAACCAAG	5'- phosphorylation
		PZ 24	GTGTGTCAATGTACACCATCCTGCTGTTTTCATGTTACAACCCATCAGAG	
TL17	pNZnisA-E3-his2	PZ 5	AATGTATGCCAAGTTGTAACTAAGCTTTCTTTGAACCAAAATTAGAAAACCAAG	5'- phosphorylation
		PZ 25	CTACTGTCAATGTACAATGACAAGTTGCTGTTTTCATGTTAC	
TL18	pNZnisA-E3-his2	PZ 7	GAATGTATGGCTTGGTGTAAATAAGCTTTCTTTGAACCAAAATTAGAAAACCAAG	5'- phosphorylation
		PZ 26	GTGTGTCAATGTACAATGACAAGTTGCTGTTTTCATGTTAC	
TL19	pNZnisA-E3-his2	PZ 5	AATGTATGCCAAGTTGTAACTAAGCTTTCTTTGAACCAAAATTAGAAAACCAAG	5'- phosphorylation
		PZ 27	CTACTGTCAATGTACAATAATGACAAGTTGCTGTTTTCATGTTAC	
TL20	pNZnisA-E3-his2	PZ 7	GAATGTATGGCTTGGTGTAAATAAGCTTTCTTTGAACCAAAATTAGAAAACCAAG	5'- phosphorylation
		PZ 28	GTGTGTCAATGTACACCAATGACAAGTTGCTGTTTTCATGTTAC	

Table S1 Primers for PCRs used in this study (continuing).

Mutants	Templates	primers	Nucleic acid sequences (5' to 3')	Characteristics (5'-lable)
Nisin(2D)	pNZnisA-E3-his2	PZ29	AATGCGTGGTGATGCACCTG	5'- phosphorylation
		PZ30	GATAGTATTTCGCTATGTACACCCGGTTG	
Nisin(2E)	pNZnisA-E3-his2	PZ29	AATGCGTGGTGATGCACCTG	5'- phosphorylation
		PZ31	GAAAGTATTTCGCTATGTACACCCGGTTG	
TL19(2D)	TL19	PZ29	AATGCGTGGTGATGCACCTG	5'- phosphorylation
		PZ30	GATAGTATTTCGCTATGTACACCCGGTTG	
TL19(34A)	TL19	PZ32	CATGTATGCCAAGTTGTAACTAAGCTTTCTTTGAACCAAAATTAGAAAACCAAG	5'- phosphorylation
		PZ 27	CTACTGTCAATGTACAATAATGACAAGTTGCTGTTTTCATGTTAC	
TL19(2D, 34A)	TL19(2D)	PZ32	CATGTATGCCAAGTTGTAACTAAGCTTTCTTTGAACCAAAATTAGAAAACCAAG	5'- phosphorylation
		PZ 27	CTACTGTCAATGTACAATAATGACAAGTTGCTGTTTTCATGTTAC	
Sequencing primer		PrXZ12	CTATCAATCAAAGCAACACGTGC	
